# A systematic comparison of recurrent event models for application to composite endpoints

**DOI:** 10.1186/s12874-017-0462-x

**Published:** 2018-01-04

**Authors:** Ann-Kathrin Ozga, Meinhard Kieser, Geraldine Rauch

**Affiliations:** 1 Insititue of Medical Biometry and Epidemiology, University Medical Center Hamburg-Eppendorf, Martinistraße 52, Hamburg, 20246 Germany; 2 Institute of Medical Biometry and Informatics, Universtiy Medical Center Ruprecht-Karls Universtiy Heidelberg, Im Neuenheimer Feld 130.3, Heidelberg, 69120 Germany; 3 Charité - Universitätsmedizin Berlin, corporate member of Freie Universität Berlin, Humboldt-Universität zu Berlin, Berlin Institute of Health, Institute of Biometry and Clinical Epidemiology, Charitéplatz 1, Berlin, 10117 Germany

**Keywords:** Recurrent event analysis, Composite endpoints, Simulation study

## Abstract

**Background:**

Many clinical trials focus on the comparison of the treatment effect between two or more groups concerning a rarely occurring event. In this situation, showing a relevant effect with an acceptable power requires the observation of a large number of patients over a long period of time. For feasibility issues, it is therefore often considered to include several event types of interest, non-fatal or fatal, and to combine them within a composite endpoint. Commonly, a composite endpoint is analyzed with standard survival analysis techniques by assessing the time to the first occurring event. This approach neglects that an individual may experience more than one event which leads to a loss of information. As an alternative, composite endpoints could be analyzed by models for recurrent events. There exists a number of such models, e.g. regression models based on count data or Cox-based models such as the approaches of Andersen and Gill, Prentice, Williams and Peterson or, Wei, Lin and Weissfeld. Although some of the methods were already compared within the literature there exists no systematic investigation for the special requirements regarding composite endpoints.

**Methods:**

Within this work a simulation-based comparison of recurrent event models applied to composite endpoints is provided for different realistic clinical trial scenarios.

**Results:**

We demonstrate that the Andersen-Gill model and the Prentice- Williams-Petersen models show similar results under various data scenarios whereas the Wei-Lin-Weissfeld model delivers effect estimators which can considerably deviate under commonly met data scenarios.

**Conclusion:**

Based on the conducted simulation study, this paper helps to understand the pros and cons of the investigated methods in the context of composite endpoints and provides therefore recommendations for an adequate statistical analysis strategy and a meaningful interpretation of results.

**Electronic supplementary material:**

The online version of this article (10.1186/s12874-017-0462-x) contains supplementary material, which is available to authorized users.

## Background

In many clinical trials, the comparison of a rarely occurring event between different treatment groups is of primary interest. To demonstrate a relevant effect and reach an acceptable power, a high number of patients has to be included in the study and observed for a long time period. This can be avoided by considering not only one type of event but several different event types of clinical interest which can be combined within a so-called composite endpoint. Thereby, the expected number of events increases and, as a consequence, the power increases as well. The components of a composite endpoint ideally correspond to the same treatment effect; however this often is not the case in clinical application. The most common and also most simple approach to analyze a composite endpoint is to investigate the time to the first event by the common Cox model [1]. The resulting treatment effect also denoted as the all-cause hazard ratio has the advantage that it has a rather intuitive interpretation from a clinical perspective as only the direct effect of a treatment is measured [2]. However, one obviously neglects that an individual may experience more than one non-fatal event which leads to a loss of information. Including recurrent events to quantify the treatment effect seems appealing as the information from each patient is fully exhausted. On the other hand, the different event processes are usually rather complex and as a consequence a corresponding effect measure will be a mixture of the treatment’s direct and indirect effects making the interpretation more difficult [2]. Whereas the time to first event approach defines the current standard and is therefore already well understood, the application of recurrent event models to composite endpoints is rather rare. The aim of this work therefore is to evaluate the performance of existing recurrent event models for the specific data situation of a composite endpoint which is commonly characterized by the following properties:

- For each event type, recurrent or terminal, there exist separate event processes that might be correlated or not.
- The event-specific treatment effects related to the different event types may deviate.
- After occurrence of an event, the instantaneous baseline risk for a subsequent event, fatal or non-fatal, increases.
- The instantaneous risk for a subsequent event depends on the time when the previous event occurred.
- After occurrence of an event, the relative treatment effect for a subsequent event (in terms of the hazard ratio) may change.

The most simple analysis approach in a recurrent event setting is to count the events observed within a given time period. These counts may, for example, follow a Poisson, a quasi-Poisson or a negative binomial distribution [3].

Whenever patients are not all fully observed but are subject to an underlying censoring mechanism, analysis strategies for event times should be preferred over simple counting approaches. As this situation is much more common in clinical application, our focus lies on models for event times rather then on counting models. The most frequently applied analysis method for recurrent time-to-event data is the model by Andersen and Gill [4] which is based on the common Cox proportional hazards model [1]. The Andersen-Gill model assumes independence between all observed event times irrespective whether these event times correspond to the same patient or to different patients. Two other (stratified) Cox-based conditional models were proposed by Prentice, Williams, and Peterson [5] which incorporate the order of events. These two approaches are based on different time scales, the gap time and the total time scale. The gap time approach investigates the time since the last event whereas the calendar or total time scale considers the time since study entry. As a further alternative, an unconditional marginal model was proposed by Wei, Lin, and Weissfeld [6]. This model ignores the order of occurrence of the events. Therefore, for each subsequent event all individuals are at risk independent of a proceeding event. The model by Wei et al. [6] is also based on a total time scale.

All these models can be extended by frailty terms to model individual patients’ heterogeneity in the baseline hazards [7–9]. For the Anderson-Gill model, Lin and Wei [10] proposed a robust variance estimator to account for individual patients’ heterogeneity.

All models introduced above are originally formulated to model a single-event process. The situation of several correlated or independent event processes related to different event types is not taken into account. Rogers et al. [11], Mazroui et al. [12], and Rondeau et al. [13] also looked at the joint frailty model which connects one or two recurrent event processes with another process leading to a fatal event through an individual frailty. Additionally, several event time processes (non-fatal and fatal) can be displayed by a multi-state model with equal or different transition hazards between the events [8, 14, 15].

Some of the above models have been systematically compared before [3, 11, 16–18]. However, the methods were not investigated in the special context of composite endpoints as described above. Rogers et al. [11] considered in their simulations a recurrent event and a dependent fatal event but did not account for a change in the hazard ratio after occurrence of a first event.

In this paper, we focus on a comparison between the common Anderson-Gill model [4], the models by Prentice, Williams and Peterson [5], and the model from Wei, Lin and Weissfeld [6]. We investigate different data settings with two event processes, one recurrent non-fatal event and a fatal event. The comparison is based on the statistical properties of the models’ treatment effect estimator and its correct interpretation, on the underlying model assumptions, and on the robustness under deviations from these assumptions. The aim is to deduce recommendations for the choice of an appropriate analysis model which addresses the specific data structure of clinical trials with composite endpoints. The performance properties of the different models will be investigated using Monte-Carlo simulations based on realistic clinical trial settings. The paper is organized as follows: We will start with an introduction of the general framework and the different models in the next section. In the section the simulation study for a performance comparison of the different methods is described. Afterwards the results are presented. We discuss our methods and results and finally we finish the article with concluding remarks.

## Modeling recurrent events

**Fig. 1:**
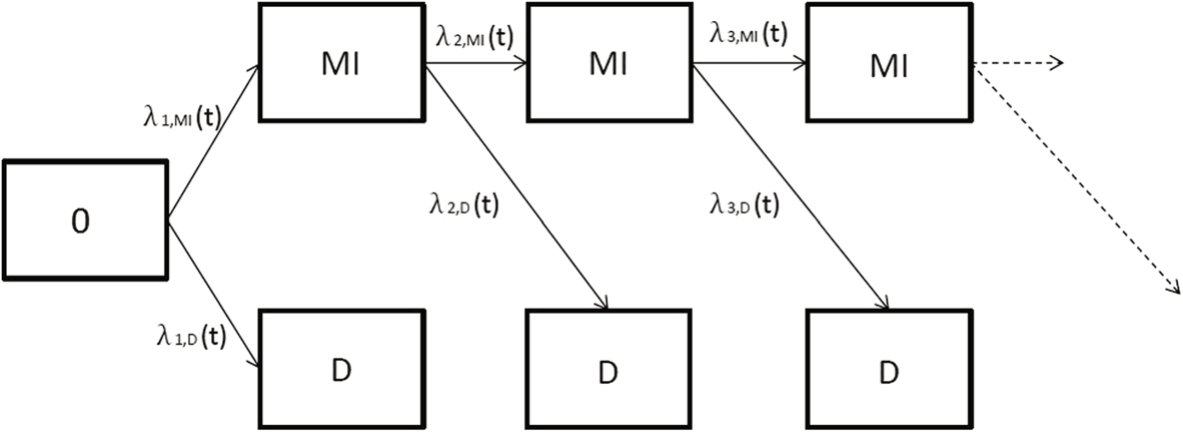
Multi-state model displaying the event process for a non-fatal (MI=myocardial infarction) and a fatal event (D=death); *λ*_*j*,*MI*_(*t*), *λ*_*j*,*D*_(*t*) are the transition hazards at time *t* for the *j*th event

Within this work, we consider a randomized, controlled, two-armed clinical trial with a composite primary endpoint which is composed of two different event types. Let us assume that there is one non-fatal, possibly recurrent event, say myocardial infarction (MI), and one fatal event like death (D). A total of *n* individuals are allocated in a 1:1 ratio to the experimental group (E) and to the control (C). The group allocation of individual *i* is expressed by the covariate *X*_*i*_ which equals 1 whenever the patient belongs to the experimental group and 0 otherwise. Each individual *i*=1,…,*n* can experience up to *j*=1,…,*k* events of the same or of differing types. Thereby, *k* which is the maximal number of considered events per patient is restricted here for the sake of simplicity. The process for the event occurrences can be described by a so-called multi-state model, compare [8, 14, 15]. An individual enters the study at an initial state 0. Every time an event occurs, the individual leaves the previous state and enters a new event state. If this observed event is non-fatal, the individual can experience more subsequent non-fatal events or the fatal event. The instantaneous risk to experience a *j*th event of type MI or D at time *t* given that the individual has experienced *j*−1 non-fatal events before is given by the transition hazards *λ*_*j*,*MI*_(*t*) and *λ*_*j*,*D*_(*t*), respectively. Figure 1 displays the corresponding multi-state model.

Most approaches to analyze recurrent events are extensions of the well-known Cox model [1], which is shortly presented in the following. Moreover, we will introduce several extensions of the standard Cox model such as the model from Andersen and Gill [4], the models from Prentice, Williams and Peterson [5], as well as the model from Wei, Lin and Weissfeld [6]. Subsequently, these models will be systematically compared for the special data situations met in the context of a clinical trial with a composite primary endpoint.

### Cox proportional hazards model

The Cox proportional hazards model is the most common approach to assess a treatment effect for time-to-event data between two or more groups with or without further covariates [1]. For a clinical trial with two treatment groups and no further covariates, as described above, the hazard for an individual *i* is modeled as

1 $$ \lambda_{i}(t)=\lambda_{0}(t)\exp(\beta X_{i}),\ i=1,{\ldots},n,  $$

where *λ*_0_(*t*) refers to a common baseline hazard and *λ*_*i*_(*t*) is the hazard of individual *i* to experience an event at time *t*. The Cox model aims at estimating the coefficient *β*, where exp(*β*) refers to the hazard ratio expressing the treatment effect. The baseline hazard *λ*_0_(*t*) remains unspecified implying that the Cox model is semiparametric. The hazard ratio exp(*β*) is assumed to be constant over time which means that the hazards related to the groups are proportional. The coefficient *β* is estimated by solving the partial likelihood-function

2 $$ L(\beta)=\prod_{i=1}^{n} {\left(\frac{exp(\beta X_{i})}{\sum_{l\in R^{Cox}(T_{i})}{exp(\beta X_{l})}}\right)^{\delta_{i}}},  $$

where *T*_*i*_, *i*=1…,*n*, are the individual-specific distinct event times and *δ*_*i*_ is the event indicator which equals 1 for an event and 0 for censoring. The risk set *R*^*Cox*^(*t*) indicates the number of individuals that are at risk for an event just prior to time point *t*, meaning all individuals that are neither censored nor did they experience an event just prior to *t*. The risk set is thus defined as

3 $$\begin{array}{@{}rcl@{}} R^{Cox}(t):=\{l,\ l=1,{\ldots},n:T_{l}\geq t\}. \end{array} $$

The common Cox model only considers the time until the first occurring event meaning that all events after the first are neglected. When applied to a composite endpoint, this first event might correspond to different event types (here: either MI or D). The corresponding hazard *λ*_*i*_(*t*) is then referred to the all-cause hazard which is defined as the instantaneous risk to experience an event of any type at time *t* given that no event occurred before. The all-cause hazard is given as the sum over the cause-specific hazards related to each event type. For the situation considered here, this means that *λ*_*i*_(*t*)=*λ*_*iMI*_(*t*)+*λ*_*iD*_(*t*). The resulting treatment effect is given by the all-cause hazard ratio denoted as *θ*_*AllCause*_=*exp*(*β*) which is the ratio of the all-cause hazards of the experimental and the control group. The all-cause hazard ratio estimated via the common Cox model is the most commonly applied treatment effect estimator to analyze composite endpoints. This strategy can thus be regarded as the reference procedure.

As indicated above, a serious shortcoming of this approach is that all events occurring after the first are neglected. This leads to a loss in information and power. However, the common Cox model can be extended to model recurrent events in different ways as described in the following.

### Andersen-Gill model

The Andersen-Gill model is probably the most often applied model for recurrent event times and is a simple extension of the Cox model [4]. It is based on the assumption that the instantaneous risk to experience an event at time *t* since study entry remains the same irrespective of the fact whether previous events occurred or not. This assumptions implies that the recurrent events are assumed to be independent which corresponds to a very strong assumption. If this strong assumption is fulfilled, the all-cause hazard can be estimated by using the event times of every observed event. Thus, a single patient contributes more than one piece of information depending on the number of individually observed events. The Andersen-Gill model therefore aims at estimating the same quantity as the common Cox model given by the all-cause hazard ratio *θ*_*AllCause*_. However, the estimation is based on more information as an individual who has experienced an event remains under risk for further events. This implies that the corresponding partial likelihood (2) is based on a higher number of events and on a modified risk set

4 $$\begin{array}{*{20}l} R^{AG}(t):=&\{l,\ l=1,{\ldots},n: \exists \ j\in\left\{1,{\ldots},k_{l}\right\}\ \\  &\text{with}\ T_{lj}\geq t\}, \end{array} $$

were *T*_*lj*_ are the distinct event times for individual *l*, *l*=1,…,*n*, and for the *j*th occurring event *j*=1,…,*k*_*l*_, with *k*_*l*_ being the individual-specific number of distinct observed event times, where *k*_*l*_≤*k*, *l*=1,…,*n*, is assumed meaning that the maximal number of events which are taken into account is given by *k*.

If the assumption of independent recurrent event times is not fulfilled, the Anderson-Gill model might still be applied but no longer estimates the all-cause hazard ratio. Instead, the resulting treatment effect estimator is given as a hazard ratio combining direct and indirect effects [19]. The mixed effect resulting from the Anderson-Gill model will be denoted as *θ*_*MixAG*_. This treatment effect cannot easily be parametrized and might therefore be considered as difficult for interpretation.

Applying the Andersen-Gill model is straightforward with standard statistical software by using the Cox model but with a data frame that includes all events for an individual and therefore comprises more than one entry for each individual. A step-by-step introduction for applying the Andersen-Gill model to a small exemplary data set in the software R [20] is provided in the Additional file 1.

### Prentice-Williams-Peterson models

Prentice, Williams, and Peterson [5] describe in their work two approaches to ‘relate the hazard function to preceding failure time history’. These methods are stratified Cox-based approaches where the first considers the time since study entry (total time or calendar time scale) while the other incorporates the time since the previous event (gap time scale). If again a clinical trial with two treatment groups and no further covariates is considered, in the total time approach the hazard for an individual *i* for the *j*th recurrent event is modeled as

5 $$\begin{array}{*{20}l} &\lambda_{ij}(t)=\lambda_{0j}(t)\exp(\beta_{j} X_{ij}), \\  & i=1,{\ldots},n, \ j=1,{\ldots},k_{i},\ k_{i}\leq k, \end{array} $$

whereas in the gap time approach the hazard is modeled as

6 $$\begin{array}{*{20}l} &\lambda_{ij}(t)=\lambda_{0j}(t-t_{j-1})\exp(\beta_{j} X_{ij}),\\ & i=1,{\ldots},n, \ j=1,{\ldots},k_{i},\ k_{i}\leq k. \end{array} $$

It can be seen that the underlying model is similar to the common Cox model (1) but for each recurrent event *j*=1,…,*k*_*i*_ a separate hazard function is modeled with an own baseline hazard *λ*_0*j*_ and a regression parameter *β*_*j*_. Thus, the hazards for a recurrent event may change after a previous event meaning that the current risk to experience an event can be influenced by the previous events. The order number *j* of an event defines a stratification variable within theses approaches, so that in stratum 1 there are all first event times, in stratum 2 there are all second event times, and so on. An individual is at risk for the *j*th event only if it experienced a previous (*j*−1)th event. Thus, in this two models the hazard at time *t* for the *j*th recurrence are conditional on the entire previous events. The partial likelihood can be written as a product of the strata-specific partial likelihoods

7 $$ L(\beta)=\prod_{j=1}^{k}{L_{j}}(\beta),  $$

with

8 $$ L_{j}(\beta)=\prod_{i=1}^{n}{\left(\frac{exp\left(\beta_{j} X_{ij}\right)}{\sum_{l \in R^{PWP}_{j}\left(T_{ij}\right)}{exp\left(\beta_{j} X_{l}\right)}}\right)^{\delta_{ij}}}.  $$

The risk sets are defined separately for each stratum. For the total time model, the risk set is given as

9 $$\begin{array}{@{}rcl@{}} R^{PWP}_{j}(t):=\left\{l,\ l=1,{\ldots},n:T_{l(j-1)}<t\leq T_{lj}\right\}, \end{array} $$

whereas for the gap time model the risk set is

10 $$\begin{array}{@{}rcl@{}} R^{PWP}_{j}(t):=\left\{l,\ l=1,{\ldots},n:(T_{lj}-T_{l(j-1)})\geq t\right\}, \end{array} $$

where again *T*_*lj*_ are the distinct event times for individual *l*, *l*=1,…,*n*, and for the *j*th occurring event *j*=1,…,*k*_*l*_, *k*_*l*_≤*k*, *l*=1,…,*n*.

It should be noted that the maximal number of recurrent events for an individual patient given by *k* determines the number of strata. The models by [5] can be applied to estimate strata-specific treatment effects *β*_*j*_, *j*=1,…,*k*. However, when analyzing a composite endpoint, one is usually interested in a single treatment effect estimator quantifying the net effect. Setting *β*_1_=*β*_2_=…=*β*_*k*_=*β* within the above partial likelihood allows to estimate a common parameter *β*. The corresponding treatment effect in terms of a hazard ratio exp(*β*) also corresponds to a mixed effect denoted as *θ*_*MixPWP*_.

The implementation can again be easily conduced by adapting the standard Cox model. For the Prentice-Williams-Peterson total time approach the same data structure as in the Andersen-Gill model is required but with an additional stratum variable which counts the number of events for each individual. For the gap time model all starting times are set to zero and the stopping time denotes the time since the previous event. A corresponding R code is again provided in the Additional file 1.

### Wei-Lin-Weissfeld model

The model by Wei, Lin, and Weissfeld [6] is also a stratified Cox-based approach where the strata are defined as described in the previous section for the Prentice-Williams-Peterson models. The hazard function is modeled equivalently to the Prentice-Williams-Peterson total time model as given in (6).

In contrast to the models by [5], an individual is at risk for every (recurrent) event as long as it is under observation. As a consequence, an individual is at risk for a subsequent event even if no previous event occurred. Thus, the dependence structure between the events observed for an individual is not specified. Strata-specific effect estimators *β*_*j*_, *j*=1,…,*k*, can be obtained from the partial likelihoods (8) with the strata-specific risk sets defines as

11 $$\begin{array}{*{20}l} R^{WLW}_{j}(t):=&\{l,\ l=1,{\ldots},n:\exists \ j\in\{1,{\ldots},k\} \ \\ &  \text{with}\ T_{lj}\geq t\}, \end{array} $$

where *T*_*lj*_ are the event times for individual *l*, *l*=1,…,*n*, and for the *j*th occurring event *j*=1,…,*k*. However, in contrast to the definition above, if the number of observed events for an individual *k*_*l*_ is smaller than the maximal number of counted events *k*, ‘artificial’ event times $\phantom {\dot {i}\!}T_{lj}:=T_{lk_{l}},\ j>k_{l}$, are defined with an event indicator *δ*_*lj*_ that equals 0 for these cases.

A combined average regression coefficient is obtained by means of a simple linear combination of the strata-specific parameters, e.g. $\beta :=1/ k\cdot \sum _{j=1}^{k}\beta _{j}$. Because the strata-specific regression coefficients are usually correlated, a correlation-adjusted variance estimator for this average treatment effect can be obtained as described in [6]. The resulting treatment effect in terms of the hazard ratio exp(*β*) again corresponds to a mixed effect denoted as *θ*_*MixWLW*_. Note that the more strata there are under consideration, the less individuals will remain per strata which results in a strata-specific effect estimation with low precision. Therefore, when applying the Wei-Lin-Weissfeld model, the number of events per patient to be considered in the analysis should be limited depending on the overall number of patients. For the simulations performed within this work we considered strata for every observed event in order to assess the impact of this decreasing precision.

As before, the Wei-Lin-Weissfeld model can be implemented by means of the Cox model. The data structure must be given by only one time variable which describes all event times. Each individual is represented in each stratum where artificial event times are generated if the individual does not experience the maximal number of events taken into account as described before. An explanatory implementation in the software R is given in the Additional file 1.

## Methods

In order to provide a systematic comparison between the models introduced before within the context of composite endpoints, a simulation study with the software R was performed [20]. Various data scenarios considering a composite endpoint composed of two independent event processes were investigated corresponding to a non-fatal recurrent event and a fatal event. Generally, composite endpoints are usually composed of more than one recurrent event either with or without incorporating an additional fatal event, e.g. myocardial infarction, stroke, unstable angina, and specific causes of death [21, 22]. Furthermore, the different event processes are often correlated in practice and in addition there can be fatal events which are not influenced by the treatment under investigation. The latter situation corresponds to a competing risk scenario where the competing event is not treatment-related. However, our intention was to investigate a rather simple example to better understand the basic performance properties of the models. The results of our work define the basis to investigate the models’ performance under more complex event processes in future work.

We investigated the magnitude of the models’ treatment effect estimators along with the corresponding power values. As we investigate the models’ performance for event times resulting from two independent event processes, the model assumptions can be fulfilled for each event process separately whereas the joint event times which do not differentiate between the event types do no longer fulfill these assumptions. Therefore, the true treatment effects for the composite endpoint which are estimated by the different models cannot be parametrized. It is therefore not possible to quantify a potential bias of the point estimators for the treatment effects but only to compare the estimators resulting from the different models.

The robustness of the different models was evaluated by considering simulation scenarios that violated the underlying model assumptions for each event process separately. As motivated in the introduction, the typical data situation of a composite endpoint is commonly characterized by a dependence of instantaneous baseline hazard and the relative treatment effect on time and/or on the time point of a previous event. The hazards for the recurrent event myocardial infarction (MI) and the fatal event death (D) are therefore modeled as

12 $$\begin{array}{*{20}l} & \lambda_{ij}^{MI}(t)= \lambda_{0j}^{MI}\left(t,t_{prev}\right) exp\left(\beta^{MI}\left(t_{prev}\right)\cdot X_{ij} \right), \\ & i=1,{\ldots},n,\ j=1,{\ldots},k_{i},\ k_{i}\leq k, \end{array} $$

and

13 $$\begin{array}{*{20}l} &\lambda_{i}^{D}(t)=\lambda_{0}^{D}\left(t,t_{prev}\right) exp\left(\beta^{D}\left(t_{prev}\right)\cdot X_{i}\right), \\  & i=1,{\ldots},n. \end{array} $$

Thereby, $\lambda _{0j}^{\ast }(t,t_{prev})$ denotes the baseline hazard and *exp*(*β*^∗^(·)) the treatment effect in terms of the hazard ratio.

Five main simulation scenarios are considered. The investigated hazard functions and hazard ratios for these scenarios are displayed in Table 1. The hazard functions and underlying parameters were chosen such that a reasonable but rather small number of events is expected to be observed within the observational period.

**Table 1:** Simulation scenarios

Scenario	$\lambda _{0}^{MI}(t,t_{prev})$	$\lambda _{0}^{D}(t,t_{prev})$	*exp*(*β*^*MI*^(*t*_*prev*_))	*exp*(*β*^*D*^(*t*_*prev*_))
1*a*	0.25	0.25	0.5	0.5
1*b*	0.25	0.25	0.5	0.7
1*c*	0.25	0.25	0.7	0.5
1*d*	0.25	0.25	0.7	1.5
1*e*	0.25	0.25	1.5	0.7
2*a*	$0.25\cdot 1/\sqrt {t_{prev}}$	$0.25\cdot 1/\sqrt {t_{prev}}$	0.5	0.5
2*b*	$0.25\cdot 1/\sqrt {t_{prev}}$	$0.25\cdot 1/\sqrt {t_{prev}}$	0.5	0.7
2*c*	$0.25\cdot 1/\sqrt {t_{prev}}$	$0.25\cdot 1/\sqrt {t_{prev}}$	0.7	0.5
2*d*	$0.25\cdot 1/\sqrt {t_{prev}}$	$0.25\cdot 1/\sqrt {t_{prev}}$	0.7	1.5
2*e*	$0.25\cdot 1/\sqrt {t_{prev}}$	$0.25\cdot 1/\sqrt {t_{prev}}$	1.5	0.7
3*a*	*t* ^0.3^	*t* ^0.3^	0.5	0.5
3*b*	*t* ^0.3^	*t* ^0.3^	0.5	0.7
3*c*	*t* ^0.3^	*t* ^0.3^	0.7	0.5
3*d*	*t* ^0.3^	*t* ^0.3^	0.7	1.5
3*e*	*t* ^0.3^	*t* ^0.3^	1.5	0.7
3*f*	1.5·*t*^0.3^	*t* ^0.3^	0.5	0.5
4*a*	0.25	0.25	0.5 exp(0.05*ln*(0.5)·*t*_*prev*_)	0.5 exp(0.05*ln*(0.5)·*t*_*prev*_)
4*b*	0.25	0.25	0.5 exp(0.05*ln*(0.5)·*t*_*prev*_)	0.7 exp(0.05*ln*(0.7)·*t*_*prev*_)
4*c*	0.25	0.25	0.7 exp(0.05*ln*(0.7)·*t*_*prev*_)	0.5 exp(0.05*ln*(0.5)·*t*_*prev*_)
4*d*	0.25	0.25	0.7 exp(0.05*ln*(0.7)·*t*_*prev*_)	1.5 exp(0.05*ln*(1.5)·*t*_*prev*_)
4*e*	0.25	0.25	1.5 exp(0.05*ln*(1.5)·*t*_*prev*_)	0.7 exp(0.05*ln*(0.7)·*t*_*prev*_)
5*a*	0.25	0.25	0.5 exp(−0.05*ln*(0.5)·*t*_*prev*_)	0.5 exp(−0.05*ln*(0.5)·*t*_*prev*_)
5*b*	0.25	0.25	0.5 exp(−0.05*ln*(0.5)·*t*_*prev*_)	0.7 exp(−0.05*ln*(0.7)·*t*_*prev*_)
5*c*	0.25	0.25	0.7 exp(−0.05*ln*(0.7)·*t*_*prev*_)	0.5 exp(−0.05*ln*(0.5)·*t*_*prev*_)
5*d*	0.25	0.25	0.7 exp(−0.05*ln*(0.7)·*t*_*prev*_)	1.5 exp(−0.05*ln*(1.5)·*t*_*prev*_)
5*e*	0.25	0.25	1.5 exp(−0.05*ln*(1.5)·*t*_*prev*_)	0.7 exp(−0.05*ln*(0.7)·*t*_*prev*_)
5*f*	0.25	0.25	0.5 exp(−0.5*ln*(0.5)·*t*_*prev*_)	0.5 exp(−0.5*ln*(0.5)·*t*_*prev*_)

$\lambda _{0}^{MI}(t,t_{prev})$ baseline hazard function for the recurrent event (myocaridal infarction); $\lambda _{0}^{D}(t,t_{prev})$ baseline hazard function for the fatal event (death); *exp*(*β*^*MI*^(*t*_*prev*_)) hazard ratio for the recurrent event (myocardial infarction); *exp*(*β*^*D*^(*t*_*prev*_)) hazard ratio for the fatal event (death)

Scenarios 1*a*−1*e* mimics the situation that the hazard and the hazard ratio do not change after occurrence of an event. However, the treatment effect can differ between the fatal event (D) and the recurrent event (MI) with either equal or opposite effect directions. Scenarios 2 capture a change in the baseline hazard either dependent on the previous event time (Table 1, Scenarios 2*a*−2*e*) or on the current time (Table 1, Scenarios 3*a*−3*f*). This change in the baseline hazard can be accounted for with the stratified models (Prentice-Williams-Peterson and Wei-Lin-Weissfeld) but not with the Andersen-Gill model. Whereas for Scenarios 3*a*−3*e*, the baseline risks are time-dependent but equal for both event types, Scenario 3*f* reflects the common situation where the baseline risks for the recurrent event is higher than for the fatal event [22]. Finally, Scenarios 4 and 5 illustrate situations where a previous event also influences the relative treatment effect in terms of the hazard ratio. We assume this change in the hazard ratio to be dependent on the previous event time with a systematically increasing effect (Table 1, Scenarios 4*a*−4*e*) or a systematically decreasing effect (Table 1, Scenarios 5*a*−5*f*) depending on the previous event time. Thereby, the baseline risks as well as the starting treatment effects at time 0 remain the same for both event types as in the other investigated scenarios. Note that Scenario 5*f* is equal to Scenario 5*a* but the ’decreasing factor’ is particularly large in magnitude. Therefore, this scenario illustrates a situation were the hazard ratio very strongly depends on the previous event time. Throughout all Scenarios 1−5, the hazard ratio is the same for the fatal and the non-fatal event in Scenario *a* whereas Scenario *b* corresponds to situations with a higher effect for the recurrent event but the effects pointing into the same direction. This is a situation most commonly met in clinical applications, compare [21, 22]. For Scenario *c* the effects again point into the same direction but with a greater effect for the fatal event. Scenarios *d* and *e* reflect situations where the event-specific effects point into opposite directions with a negative treatment effect for the fatal event and a positive effect for the recurrent event in Scenarios *d* and vice versa in Scenarios *e*.

Bender et al. [23] described in general how non-recurrent event times can be simulated, and Jahn-Eimermacher et al. [24] followed their approach and developed an algorithm to recursively simulate recurrent event times in a total time model. We base our simulations on both methods as we consider at fatal non-recurrent and a non-fatal recurrent event. The event times for the fatal event were simulated according to [23] with the restriction that if a non-fatal event has already occurred the baseline hazard and the hazard ratio are allowed to change based on the previous event time (compare the definition of the hazard function for death (13)). For the recurrent event, we started by simulating gap times as described in [24]. To allow a change in the hazard and hazard ratio, the gap times were altered depending on the time or time point of the previous event. For the models based on total event times, these are generated by summing up all observed gap times, where the individual total time for the first event corresponds to the first individual gap time. Note that as the gap times follow different distributions, the distribution of the actual total event times is not identifiable. The simulated individual total time for the recurrent event is censored whenever it exceeds the simulated death time or the individual follow-up time. The simulated individual total time for the fatal event is censored if it exceeds the individual follow-up time. The individual follow-up times were simulated with uniformly distributed entry times within the interval [0,1] and a minimal follow-up of 2 years. We additionally investigated minimal follow-up times of 5 and 10 years for some specific data scenarios.

For each scenario, we simulated a total of 5000 data sets each with 200 patients in total (i.e. 100 patients per group). Subsequently, all models described above are applied to the simulated data sets. Thereby, the strata-specific approaches use a number of strata which is given by the maximal number of observed events per individual.

## Results

Table 2 presents the average values and corresponding standard deviations of individual-specific observed number of events, the estimated hazard ratio derived from the Andersen-Gill model ($\hat \theta _{MixAG}$), the estimated hazard ratios derived from the two models of Prentice, Williams and Peterson (total time model: $\hat \theta _{MixPWP1}$, gap time model: $\hat \theta _{MixPWP2}$), and the estimated hazard ratio from the Wei-Lin-Weissfeld model ($\hat \theta _{MixWLW}$) along with the corresponding empirical power values.

**Table 2:** Simulation results

Simulation parameters					Mean (SD)	$\hat \theta $ ($sd(\hat \theta)$), *power*			
Sc.	$\lambda _{0}^{MI}(t,t_{prev})$	$\lambda _{0}^{D}(t,t_{prev})$	*exp*(*β*^*MI*^(*t*_*prev*_))	*exp*(*β*^*D*^(*t*_*prev*_))	*#* events	$\hat \theta _{MixAG}$	$\hat \theta _{MixPWP1}$	$\hat \theta _{MixPWP2}$	$\hat \theta _{MixWLW}$
1*a*	0.25	0.25	0.5	0.5	2.93 (0.65)	0.50 (0.13), 0.83	0.50 (0.13), 0.83	0.51 (0.13), 0.83	0.46 (0.14), 0.82
1*b*	0.25	0.25	0.5	0.7	2.93 (0.65)	0.50 (0.13), 0.83	0.50 (0.13), 0.82	0.51 (0.13), 0.82	0.46 (0.14), 0.82
1*c*	0.25	0.25	0.7	0.5	3.02 (0.63)	0.70 (0.16), 0.39	0.71 (0.16), 0.38	0.71 (0.16), 0.39	0.68 (0.19), 0.37
1*d*	0.25	0.25	0.7	1.5	3.00 (0.64)	0.72 (0.17), 0.35	0.72 (0.17), 0.34	0.72 (0.17), 0.34	0.68 (0.19), 0.36
1*e*	0.25	0.25	1.5	0.7	3.66 (0.73)	1.53 (0.30), 0.57	1.52 (0.30), 0.56	1.52 (0.29), 0.56	1.71 (0.41), 0.59
2*a*	$0.25\cdot 1/\sqrt {t_{prev}}$	$0.25\cdot 1/\sqrt {t_{prev}}$	0.5	0.5	2.15 (0.43)	0.52 (0.13), 0.81	0.51 (0.13), 0.81	0.51 (0.13), 0.81	0.48 (0.14), 0.80
2*b*	$0.25\cdot 1/\sqrt {t_{prev}}$	$0.25\cdot 1/\sqrt {t_{prev}}$	0.5	0.7	2.15 (0.43)	0.52 (0.13), 0.81	0.51 (0.13), 0.81	0.51 (0.13), 0.81	0.48 (0.14), 0.80
2*c*	$0.25\cdot 1/\sqrt {t_{prev}}$	$0.25\cdot 1/\sqrt {t_{prev}}$	0.7	0.5	2.20 (0.46)	0.71 (0.16), 0.37	0.71 (0.17), 0.36	0.71 (0.17), 0.37	0.69 (0.18), 0.35
2*d*	$0.25\cdot 1/\sqrt {t_{prev}}$	$0.25\cdot 1/\sqrt {t_{prev}}$	0.7	1.5	2.18 (0.44)	0.73 (0.17), 0.32	0.72 (0.17), 0.32	0.72 (0.17), 0.32	0.70 (0.19), 0.34
2*e*	$0.25\cdot 1/\sqrt {t_{prev}}$	$0.25\cdot 1/\sqrt {t_{prev}}$	1.5	0.7	2.44 (0.65)	1.45 (0.29), 0.57	1.52 (0.31), 0.53	1.51 (0.30), 0.53	1.64 (0.33), 0.54
3*a*	*t* ^0.3^	*t* ^0.3^	0.5	0.5	5.69 (0.99)	0.47 (0.07), 1	0.48 (0.07), 1	0.48 (0.07), 1	0.38 (0.08), 0.99
3*b*	*t* ^0.3^	*t* ^0.3^	0.5	0.7	5.69 (0.99)	0.48 (0.07), 1	0.49 (0.07), 0.99	0.49 (0.07), 0.99	0.39 (0.08), 0.99
3*c*	*t* ^0.3^	*t* ^0.3^	0.7	0.5	5.83 (0.95)	0.66 (0.09), 0.91	0.66 (0.09), 0.92	0.66 (0.05), 0.93	0.60 (0.12), 0.78
3*d*	*t* ^0.3^	*t* ^0.3^	0.7	1.5	5.75 (0.97)	0.74 (0.10), 0.63	0.76 (0.10), 0.56	0.77 (0.10), 0.54	0.63 (0.12), 0.69
3*e*	*t* ^0.3^	*t* ^0.3^	1.5	0.7	7.60 (1.12)	1.43 (0.16), 0.89	1.40 (0.15), 0.87	1.39 (0.15), 0.87	1.77 (0.33), 0.85
3*f*	1.5·*t*^0.3^	*t* ^0.3^	0.5	0.5	7.52 (1.12)	0.48 (0.06), 1	0.48 (0.06), 0.99	0.48 (0.06), 1	0.35 (0.07), 0.99
4*a*	0.25	0.25	0.5*exp*(0.05*ln*(0.5)·*t*_*prev*_)	0.5*exp*(0.05*ln*(0.5)·*t*_*prev*_)	2.91 (0.65)	0.49 (0.13), 0.85	0.49 (0.13), 0.85	0.49 (0.13), 0.85	0.45 (0.13), 0.84
4*b*	0.25	0.25	0.5*exp*(0.05*ln*(0.5)·*t*_*prev*_)	0.7*exp*(0.05*ln*(0.7)·*t*_*prev*_)	2.92 (0.65)	0.49 (0.13) 0.84	0.50 (0.13), 0.84	0.50 (0.13), 0.84	0.46 (0.13), 0.84
4*c*	0.25	0.25	0.7*exp*(0.05*ln*(0.7)·*t*_*prev*_)	0.5*exp*(0.05*ln*(0.5)·*t*_*prev*_)	3.01 (0.63)	0.70 (0.16), 0.41	0.70 (0.16), 0.40	0.70 (0.16), 0.41	0.67 (0.18), 0.38
4*d*	0.25	0.25	0.7*exp*(0.05*ln*(0.7)·*t*_*prev*_)	1.5*exp*(0.05*ln*(1.5)·*t*_*prev*_)	2.99 (0.64)	0.71 (0.17), 0.37	0.71 (0.17), 0.36	0.72 (0.17), 0.36	0.67 (0.18), 0.37
4*e*	0.25	0.25	1.5*exp*(0.05*ln*(1.5)·*t*_*prev*_)	0.7*exp*(0.05*ln*(0.7)·*t*_*prev*_)	3.69 (0.73)	1.55 (0.30), 0.61	1.54 (0.30), 0.59	1.54 (0.30), 0.60	1.73 (0.42), 0.60
5*a*	0.25	0.25	0.5*exp*(−0.05*ln*(0.5)·*t*_*prev*_)	0.5*exp*(−0.05*ln*(0.5)·*t*_*prev*_)	2.95 (0.65)	0.52 (0.13), 0.80	0.52 (0.14), 0.79	0.52 (0.13), 0.79	0.48 (0.14), 0.79
5*b*	0.25	0.25	0.5*exp*(−0.05*ln*(0.5)·*t*_*prev*_)	0.7*exp*(−0.05*ln*(0.7)·*t*_*prev*_)	2.95 (0.65)	0.52 (0.13), 0.79	0.52 (0.14), 0.78	0.52 (0.14), 0.78	0.48 (0.14), 0.79
5*c*	0.25	0.25	0.7*exp*(−0.05*ln*(0.7)·*t*_*prev*_)	0.5*exp*(−0.05*ln*(0.5)·*t*_*prev*_)	3.03 (0.62)	0.71 (0.17), 0.37	0.72 (0.17), 0.37	0.72 (0.17), 0.37	0.69 (0.19), 0.35
5*d*	0.25	0.25	0.7*exp*(−0.05*ln*(0.7)·*t*_*prev*_)	1.5*exp*(−0.05*ln*(1.5)·*t*_*prev*_)	3.01 (0.63)	0.72 (0.17), 0.33	0.73 (0.17), 0.33	0.73 (0.17), 0.32	0.69 (0.19), 0.35
5*e*	0.25	0.25	1.5*exp*(−0.05*ln*(1.5)·*t*_*prev*_)	0.7*exp*(−0.05*ln*(0.7)·*t*_*prev*_)	3.61 (0.72)	1.48 (0.44), 0.62	1.47 (0.44), 0.61	1.47 (0.44), 0.61	1.65 (0.55), 0.62
5*f*	0.25	0.25	0.5*exp*(−0.5*ln*(0.5)·*t*_*prev*_)	0.5*exp*(−0.5*ln*(0.5)·*t*_*prev*_)	3.09 (0.60)	0.67 (0.24), 0.44	0.81 (0.30), 0.40	0.77 (0.33), 0.40	0.61 (0.24), 0.48

$\hat \theta $ estimated mean treatment effect; $sd(\hat \theta)$ mean standard deviation of estimated treatment effect; *power* empirical power values for the Andersen-Gill model (AG), the Prentice-Williams-Peterson total time model (PWP1), the Prentice-Williams-Peterson gap time model (PWP2), the Wei-Lin-Weissfeld model (WLW); $\lambda _{0}^{MI}(t,t_{prev})$ baseline hazard function for the recurrent event (myocaridal infarction); $\lambda _{0}^{D}(t,t_{prev})$ baseline hazard function for the fatal event (death); *exp*(*β*^*MI*^(*t*_*prev*_)) hazard ratio for the recurrent event (myocaridal infarction); *exp*(*β*^*D*^(*t*_*prev*_)) hazard ratio for the fatal event (death)

For Scenarios 1*a* to 1*e*, the baseline hazards and hazard ratios are constant in time implying that the model assumptions for the Andersen-Gill and the Prentice-Williams-Peterson approaches are fulfilled for each event process separately. It is therefore intuitive that the estimated mean hazard ratios from the Andersen-Gill and Prentice-Williams-Peterson models closely coincide thereby showing an acceptable power. For the Wei-Lin-Weissfeld approach, the magnitude of the treatment effect increases independent of the direction of the effect which is also known as ‘carry-over effect’ [25]. Remember that the Wei-Lin-Weissfeld model includes all patients in each stratum. The strata-specific effect estimators generally increase in magnitude with time as later strata in the experimental group contain more censored observations and thus the influence of a single event in the control group becomes larger resulting in an exaggerated treatment effect over time. Furthermore, the standard deviation is higher in the Wei-Lin-Weissfeld model. This can be explained by the fact that no direct global effect but strata-specific effects and variances are estimated which are combined subsequently. As later strata contain fewer events, the strata-specific standard deviation increases with time. These observations for the Wei-Lin-Weissfeld model can be generalized to most of the investigated simulation scenarios: The estimated mixed effect resulting from the Andersen-Gill model and from the models from Prentice, Williams and Peterson are nearly the same whereas the Wei-Lin-Weissfeld approach tends to result in a treatment effect of higher magnitude with a higher standard deviation. Simulation Scenarios 1*a* and 1*e* differ with respect to the constellation of the underlying effect sizes for the two event types which either point in the same or in opposite directions. The global treatment effect estimator is generally stronger influenced by the effect of the non-fatal, recurrent event. This is intuitive as the amount of non-fatal events is generally higher than the amount of fatal events and therefore the recurrent event process dominates the global treatment effect.

Simulation Scenarios 2*a* to 2*e* and 3*a* to 3*f* investigate time-dependent baseline hazards. A baseline hazard that changes with the previous event time results in only slightly differing effect estimates compared to the scenarios with a constant baseline hazard (Scenarios 2*a* to 2*e*). The standard deviation is also similar compared to Scenarios 1*a*,…,1*e* with constant baseline hazards. However, the power values decrease because the investigated baseline hazards result in fewer individuals at risk over time and, as a consequence, in a reduced number of events compared to the case of constant hazards. If the baseline hazard changes only in dependence of the time *t* (Scenarios 3*a* to 3*f*), the estimated hazard ratios from the Wei-Lin-Weissfeld model show the strongest deviations from the effect estimators of the other models. As discussed above, this is due to the so called ‘carry-over effect’.

Finally, in Scenarios 4*a* to 4*e* and 5*a* to 5*f* the treatment effect in terms of the hazard ratio changes with the previous event time. Scenarios 4*a* to 4*e* consider the situation of an effect that increases with the previous event whereas Scenarios 5*a* to 5*f* investigate a decreasing effect. The resulting treatment effect estimators for Scenarios 4*a* to 4*e* and 5*a* to 5*e* are close to the ones of Scenarios 1*a* to 1*e* considering constant hazard ratios. Here, the main influence can be observed for the power values where intuitively an increasing effect leads to a higher power whereas a decreasing effect decreases the power when compared to the results of Scenarios 1*a* to 1*e*. A difference in effect estimation between the Andersen-Gill model and the approach by Prentice, Williams and Peterson is observed if the dependence of the hazard ratio on the previous event time is extreme (Scenario 5*f*). Note that Scenario 5 illustrates a situation where the treatment effect approaches 1 with an increasing previous event time. This decrease in the magnitude of the hazard ratio over time is better captured by the conditional models of Prentice, Williams and Peterson whereas the Andersen-Gill model is much less sensitive for this situation.

We conducted additional simulations with minimal follow-up times of 5 and 10 years. The results are not shown to restrict the length of this paper. Generally, the magnitude of the estimated treatment effects also depend on the follow-up duration. With an increasing observational period, more events are observed and therefore the relation between the observed number of fatal and non-fatal events can change depending on the underlying hazard function. As a consequence, the follow-up duration especially has an influence on the effect estimator if the underlying treatment effects for the two event types point in opposite directions.

## Discussion

In this work, we investigated the performance of common recurrent event models for various data scenarios that capture different properties of composite endpoints. We considered the following situations: 1. two independent event processes for a fatal and a recurrent event with equal or differing treatment effects, 2. a change in the baseline hazard in dependence of the previous event time or the actual time, and 3. a change in the hazard ratio in dependence of the previous event time. By a Monte-Carlo simulation study, we evaluated how the recurrent event models from Andersen and Gill [4], Prentice, Williams and Peterson [5], and Wei, Lin and Weissfeld [6] perform in these situations.

Whenever the event-specific treatment effects differ, all models deliver mixed overall effects in terms of hazard ratios which cannot explicitly be parametrized as we consider two independent event processes which are combined subsequently. In our situation with one recurrent event and one fatal event, the estimated mixed hazard ratio is more influenced by the effect in the non-fatal event. This is due to the fact that the amount of recurrent events exceeds the amount of fatal events. This raises the question if it is acceptable to consider a mixed hazard ratio as a clinically relevant treatment effect measure in all data situations. A mixed effect might not be problematic if the underlying effects of the event processes are similar and point into the same direction. However, if the event-specific treatment effects point into opposite directions, interpretation of the mixed effect becomes more difficult. A small adverse effect for the fatal event can be masked by a larger positive effect for the recurrent event. This is also the case if composite endpoints are analyzed with the common Cox model with a time-to-first-event approach. However, by including all recurrent events into the analysis, the impact of the fatal non-recurrent event becomes even smaller which is important to remember when recurrent events combined with a fatal event are analyzed. Generally, it might also be of interest to investigate the models‘ performance if two or more recurrent events are considered.

For the simulation scenarios where the baseline hazards change in dependence of the actual time or the previous event time, only slight changes in the effect estimators compared to the constant baseline hazard scenarios were observed. The same holds true for the scenarios that mimic a small change in the hazard ratios dependent on the previous event time of 0.05·*t*_*prev*_. However, this is not true if a higher decreasing effect change of 0.5·*t*_*prev*_ is incorporated. In this situation the conditional models by Prentice, Williams and Peterson capture this high change in the effect better than the other models.

Throughout most of the investigated scenarios, the Andersen-Gill model and the Prentice-Williams-Peterson models show similar effect estimators, standard deviations, and power values whereas the Wei-Lin-Weissfeld model generally tends to deliver treatment effects which are larger in magnitude independent of the direction of the effect. Nonetheless, the power values of the Wei-Lin-Weissfeld model are usually smaller which is due to the considerably higher standard deviations of the estimated hazard ratios. This is due to the different definition of the risk sets within the models. For the Wei-Lin-Weissfeld approach all individuals are at risk for a subsequent event even if they did not experience the previous event and thereby the order of events is also neglected. This leads to a ‘carry-over effect’ as explained above and by [25]. Therefore, the Wei-Lin-Weissfeld model seems not the best choice to analyze a clinical trial with a composite endpoint. The differences described before between the Andersen-Gill or Prentice-Williams-Peterson models to the approach of Wei, Lin, and Weissfeld were already shown in previous works [3, 6, 16]. However, most of these previous findings are not exactly comparable to ours as the authors considered only one recurrent event process which, in contrast to most of our results, leads to greater differences between the Andersen-Gill and Prentice-Williams-Peterson approaches.

As stated above, the results from the Andersen-Gill model differ barely from the Prentice-Williams-Peterson models because the risk sets are similar for both approaches as long as only a few strata are considered in the Prentice-Williams-Peterson model. For the scenarios with a higher number of mean events, the differences between these models become more obvious which can especially be seen for Scenario 3*e* where the treatment induces more fatal events. In this case, the Andersen-Gill model remains more influenced by the recurrent event process. Furthermore, the more strongly the effect depends on the previous event time (like in scenario 5*f*) the more the effect estimates of the models by Prentice, Williams and Peterson deviate from the effect estimate by the Anderson-Gill model. This is due to the strata-specific partial likelihoods with the different risk sets for the Prentice, Williams and Peterson models. From a theoretical point of view, the Prentice-Williams-Peterson models are the only models that take the order of the events into account in the definition of the risk sets. Therefore, it seems more appropriate to use one of these conditional models instead of the method from the Andersen-Gill model.

Based on our simulation study with one fatal and one non-fatal event, the Prentice-Williams-Peterson model seems to capture most of the commonly met data scenarios for clinical trials with composite endpoints. From our results, no general recommendation regarding the choice between the total time or the gap time approach can be derived. This choice should be guided from the medical application at hand: While the total time scale usually is of interest if the disease *process* of the patient is considered as a whole, gap times might be of interest when disease *episodes* are in the medical focus.

The Wei-Lin-Weissfeld model and the Prentice-Williams-Peterson model also allow to estimate strata-specific effects which can provide an important supplementary information to better understand the magnitude of the overall mixed effect. The Wei-Lin-Weissfeld model also allows to base the analysis on alternative strata definitions. For example, separate strata for the different event types could be defined. Thereby, event-specific effect estimates could be derived by analyzing the strata-specific effects. As the order of events is neglected by this approach, this alternative strata definition cannot easily be adapted to the Prentice-Williams-Peterson models.

All these models can be extended with a frailty term to account for heterogeneity between individuals [7–9]. Irrespective of the fact whether a frailty term is explicitly modeled, robust variance estimators to adjust the variance of the corresponding effect estimator for between-subject heterogeneity should be preferred [10].

A more complex scenario would consider more than one non-fatal event, e.g. myocardial infarction, stroke, and unstable angina. These events are usually related and thus more complex frailty models which allow a correlation between event types should be investigated. Furthermore, other fatal events not related to the treatment might occur thereby inducing a competing risk scenario. We are currently working on the investigation of these models for event processes related by a frailty term to address these open topics.

## Conclusion

In conclusion, apart from the general interpretation difficulty of an overall mixed effect, the conditional models from Prentice, Williams, and Peterson [5] could be recommended to analyze clinical trials with a composite endpoint which is justified from a theoretical point of view as well as from the results of our simulation study. However, more work has to be done to consider the situation of more than two correlated event processes, e.g. myocardial infarction, stroke, and death, especially when the event-specific effects point in opposite direction. The modelling approach of correlated processes as proposed by Rogers et al. [11] could thereby be of interest.

## Additional file

Additional file 1 Entitled Additional File to the Article ’A Systematic Comparison of Recurrent Event Models for Application to Composite Endpoints’ and provides R-Code for an easy implementation of the Andersen-Gill, Prentice-Williams-Peterson, and Wei-Lin Weissfeld models as well as the Bayesian Information Criterion for the simulated scenarios. (PDF 192 kb)

## Abbreviations

AG: Andersen-Gill
C: Control
D: Death
E: Experimental
MI: Myocardial infarction
PWP: Prentice-Williams-Peterson
WLW: Wei-Lin-Weissfeld
