# Supplementary material for: A systematic comparison of recurrent event models for application to composite endpoints
Source: BMC Med Res Methodol. 2018 Jan 4;18:2. doi: 10.1186/s12874-017-0462-x (PMC5755224; doi:10.1186/s12874-017-0462-x)
Supplement: Additional file 1 — Entitled Additional File to the Article ’A Systematic Comparison of Recurrent Event Models for Application to Composite Endpoints’ and provides R-Code for an easy implementation of the Andersen-Gill, Prentice-Williams-Peterson, and Wei-Lin Weissfeld models as well as the Bayesian Information Criterion for the simulated scenarios. (PDF 192 kb) [file 12874_2017_462_MOESM1_ESM.pdf]

RESEARCH

Additional File to the Article 'A Systematic Comparison of Recurrent Event Models for Application to Composite Endpoints'

Ann-Kathrin Ozga[1,2*], Meinhard Kieser[2] and Geraldine Rauch[2,3]

*Correspondence: a.ozga@uke.de
[1] Insititue of Medical Biometry and Epidemiology, University Medical Center Hamburg-Eppendorf, Martinistraße 52, 20246 Hamburg, Germany
Full list of author information is available at the end of the article

Implementation in R

To illustrate how the analysis of recurrent event data with the models of Andersen and Gill [1], Prentice, Williams, and Peterson [2], and Wei, Lin, and Weissfeld [3] can be performed by application of the statistical software R, we will provide the corresponding program code for a small exemplary data set named *DataRec*:

Individual 1 belongs to group 1, experiences three non-fatal events at months 2, 3, 5, and is censored at month 8; individual 2 is in group 1, experiences no event and is censored at month 10: individual 3 belongs to group 2, experiences events at months 1 and 6 and is censored at months 10. We assume a common study start for each individual at time point 0.

Implementation of the Anderson-Gill model

The required data frame for the Anderson-Gill model [1] is given in Table 1.

Table 1 Data frame for AG model

ID	group	start	stop	status
1	1	0	2	1
1	1	2	3	1
1	1	3	5	1
1	1	5	8	0
2	1	0	10	0
3	2	0	1	1
3	2	1	6	1
3	2	6	10	0

Data frame as required for the Andersen-Gill model; ID: patient ID, $group$: treatment group; $start$: study entry time/previous event time; $stop$: event/censoring times; $status$: event indicator

The R code to apply the Andersen-Gill model to the data set *DataRec* is given as

$$coxph(Surv(start, stop, status) \sim group + cluster(ID), data = DataRec)$$

Thereby, the `coxph` function of the `survival` package [4] is used and `cluster(ID)` requests robust standard errors for the parameter estimates to account for individual patient heterogeneity.

Implementation of the Prentice-Williams-Peterson models

The stratified model from Prentice, Williams, and Peterson [2] based on the total time scale can be applied using the data frame as displayed in Table 2.

Table 2 Data frame for the PWP total time approach

ID	group	start	stop	status	enum
1	1	0	2	1	1
1	1	2	3	1	2
1	1	3	5	1	3
1	1	5	8	0	4
2	1	0	10	0	1
3	2	0	1	1	1
3	2	1	6	1	2
3	2	6	10	0	3

Data frame as required for the Prentice-Williams-Peterson total time model; ID: patient ID, $group$: treatment group; $start$: study entry time/previous event time; $stop$: event/censoring times; $status$: event indicator; $enum$: indicator for the stratum

The corresponding R code is given as

$$coxph(Surv(start, stop, status) \sim group{+}cluster(ID){+}strata(enum), data = DataRec)$$

The stratified model from Prentice, Williams, and Peterson [2] based on the gap time scale can be applied using the data frame as displayed in Table 3.

Table 3 Data frame for the PWP gap time approach

ID	group	start	stop	status	enum
1	1	0	2	1	1
1	1	0	1	1	2
1	1	0	2	1	3
1	1	0	3	0	4
2	1	0	10	0	1
3	2	0	1	1	1
3	2	0	5	1	2
3	2	0	4	0	3

Data frame as required for the Prentice-Williams-Peterson gap time model; ID: patient ID, $group$: treatment group; $start$: study entry time/previous event time; $stop$: event/censoring times; $status$: event indicator; $enum$: indicator for the stratum

The corresponding R code is given as

$$coxph(Surv(start, stop, status) \sim group{+}cluster(ID){+}strata(enum), data = DataRec)$$

Implementation of the Wei-Lin-Weissfeld model

The stratified model from Wei, Lin, and Weissfeld [3] can be applied using the data frame as displayed in Table 4.

Table 4 Data frame for the WLW model

ID	group	start	stop	status	enum
1	1	0	2	1	1
1	1	2	3	1	2
1	1	3	5	1	3
2	1	0	10	0	1
2	1	0	10	0	2
2	1	0	10	0	3
3	2	0	1	1	1
3	2	1	6	1	2
3	2	6	10	0	3

Data frame as required for the Wei-Lin-Weissfeld model; ID: patient ID, $group$: treatment group; $start$: study entry time/previous event time; $stop$: event/censoring times; $status$: event indicator; $enum$: indicator for the stratum

The corresponding R code is given as

$$coxph(Surv(start, stop, status) \sim group{+}cluster(ID){+}strata(enum), data = DataRec)$$

Bayesian Information Criterion for all simulated scenarios concerning the results in Table 2 of the Manuscript

In Table 5 the mean Bayesian Information Criterion [5] over 5000 simulations per scenario is given for all simulated situations listed in Table 2 of the main manuscript.

Table 5 Simulation Results: BIC

	Simulation parameters							
Sc.	$\lambda_0^{MI}(t, t_{prev})$	$\lambda_0^{D}(t, t_{prev})$	$exp(\beta^{MI}(t_{prev}))$	$exp(\beta^{D}(t_{prev}))$	$\overline{BIC}_{AG}$	$\overline{BIC}_{PWP1}$	$\overline{BIC}_{PWP2}$	$\overline{BIC}_{WLW}$
$1a$	0.25	0.25	0.5	0.5	727.93	659.21	700.38	727.73
$1b$	0.25	0.25	0.5	0.7	726.73	658.45	699.75	727.29
$1c$	0.25	0.25	0.7	0.5	831.11	750.06	797.12	828.85
$1d$	0.25	0.25	0.7	1.5	820.95	743.27	791.23	823.88
$1e$	0.25	0.25	1.5	0.7	1206.35	1061.61	1128.26	1184.12
$2a$	$0.25 \cdot 1/\sqrt{t_{prev}}$	$0.25 \cdot 1/\sqrt{t_{prev}}$	0.5	0.5	685.26	637.10	675.74	687.64
$2b$	$0.25 \cdot 1/\sqrt{t_{prev}}$	$0.25 \cdot 1/\sqrt{t_{prev}}$	0.5	0.7	684.30	636.52	675.30	687.41
$2c$	$0.25 \cdot 1/\sqrt{t_{prev}}$	$0.25 \cdot 1/\sqrt{t_{prev}}$	0.7	0.5	784.87	725.59	770.12	785.65
$2d$	$0.25 \cdot 1/\sqrt{t_{prev}}$	$0.25 \cdot 1/\sqrt{t_{prev}}$	0.7	1.5	776.49	720.20	765.71	782.26
$2e$	$0.25 \cdot 1/\sqrt{t_{prev}}$	$0.25 \cdot 1/\sqrt{t_{prev}}$	1.5	0.7	1133.92	1016.61	1087.30	1120.17
$3a$	$t^{0.3}$	$t^{0.3}$	0.5	0.5	2232.49	1809.78	1983.16	2121.24
$3b$	$t^{0.3}$	$t^{0.3}$	0.5	0.7	2200.92	1788.45	1961.25	2100.93
$3c$	$t^{0.3}$	$t^{0.3}$	0.7	0.5	2550.04	2050.49	2253.46	2404.13
$3d$	$t^{0.3}$	$t^{0.3}$	0.7	1.5	2342.49	1906.77	2106.54	2260.55
$3e$	$t^{0.3}$	$t^{0.3}$	1.5	0.7	3549.93	2672.83	3037.22	3243.98
$3f$	$1.5t^{0.3}$	$t^{0.3}$	0.5	0.5	3231.42	2443.76	2755.38	2948.67
$4a$	0.25	0.25	$0.5exp(0.05ln(0.5) \cdot t_{prev})$	$0.5exp(0.05ln(0.5) \cdot t_{prev})$	723.04	656.17	696.99	722.93
$4b$	0.25	0.25	$0.5exp(0.05ln(0.5) \cdot t_{prev})$	$0.7exp(0.05ln(0.5) \cdot t_{prev})$	721.99	655.51	696.46	722.63
$4c$	0.25	0.25	$0.7exp(0.05ln(0.7) \cdot t_{prev})$	$0.5exp(0.05ln(0.5) \cdot t_{prev})$	827.15	747.72	794.42	824.96
$4d$	0.25	0.25	$0.7exp(0.05ln(0.7) \cdot t_{prev})$	$1.5exp(0.05ln(0.5) \cdot t_{prev})$	817.47	741.24	788.97	820.49
$4e$	0.25	0.25	$1.5exp(0.05ln(1.5) \cdot t_{prev})$	$0.7exp(0.05ln(0.5) \cdot t_{prev})$	1218.22	1062.07	1136.19	1195.38
$5a$	0.25	0.25	$0.5exp(-0.05ln(0.5) \cdot t_{prev})$	$0.5exp(-0.05ln(0.5) \cdot t_{prev})$	734.93	663.46	705.21	734.66
$5b$	0.25	0.25	$0.5exp(-0.05ln(0.5) \cdot t_{prev})$	$0.7exp(-0.05ln(0.5) \cdot t_{prev})$	733.53	662.60	704.40	734.02
$5c$	0.25	0.25	$0.7exp(-0.05ln(0.7) \cdot t_{prev})$	$0.5exp(-0.05ln(0.5) \cdot t_{prev})$	835.39	752.59	800.08	833.07
$5d$	0.25	0.25	$0.7exp(-0.05ln(0.7) \cdot t_{prev})$	$1.5exp(-0.05ln(0.5) \cdot t_{prev})$	824.90	745.59	793.81	827.73
$5e$	0.25	0.25	$1.5exp(-0.05ln(1.5) \cdot t_{prev})$	$0.7exp(-0.05ln(0.5) \cdot t_{prev})$	1195.39	1048.44	1120.97	1173.75
$5f$	0.25	0.25	$0.5exp(-0.5ln(0.5) \cdot t_{prev})$	$0.5exp(-0.5ln(0.5) \cdot t_{prev})$	808.66	699.74	757.05	809.83

Mean Bayesian Information Criterion (BIC) over 5000 simulations for all corresponding simulated scenarios of Table 2 of the manuscript for the Andersen-Gill model (AG), the Prentice-Williams-Peterson total time model (PWP1), the Prentice-Williams-Peterson gap time model (PWP2), the Wei-Lin-Weissfeld model (WLW); $\lambda_0^{MI}(t, t_{prev})$ baseline hazard function for the recurrent event (myocaridal infarction); $\lambda_0^{D}(t, t_{prev})$ baseline hazard function for the fatal event (death); $exp(\beta^{MI}(t_{prev}))$ hazard ratio for the recurrent event (myocaridal infarction); $exp(\beta^{D}(t_{prev}))$ hazard ratio for the fatal event (death).

Author details

[1] Insititue of Medical Biometry and Epidemiology, University Medical Center Hamburg-Eppendorf, Martinistraße 52, 20246 Hamburg, Germany. [2] Institute of Medical Biometry and Informatics, Universtiy Medical Center Ruprecht-Karls Universtiy Heidelberg, Im Neuenheimer Feld 130.3, 69120 Heidelberg, Germany. [3]Charité - Universitätsmedizin Berlin, corporate member of Freie Universität Berlin, Humboldt-Universität zu Berlin, and Berlin Institute of Health, Institute of Biometry and Clinical Epidemiology, Charitéplatz 1, 10117 Berlin, Germany.

References

1. Andersen, P.K., Gill, R.D.: Cox's regression model for counting processes: A large sample study. The Annals of Statistics **10(4)**, 1100–1120 (1982)
2. Prentice, R.L., Williams, B.J., Peterson, A.V.: On the regression analysis of multivariate failure time data. Biometrika **68(2)**, 373–379 (1981)
3. Wei, L.J., Lin, D.Y., Weissfeld, L.: Analysis of multivariate incomplete failure time data by modeling marginal distributions. Biometrika **84(408)**, 1065–1073 (1989)
4. Therneau, T.M.: Survival Package, Software R. (2017). Version 2.41-3, https://CRAN.R-project.org/package=survival
5. Schwarz, G.: Estimating the dimension of a model. The Annals of Statistics **6(2)**, 461–464 (1978)
